# Bioimpedance-Based Heart Failure Deterioration Prediction Using a Prototype Fluid Accumulation Vest-Mobile Phone Dyad: An Observational Study

**DOI:** 10.2196/cardio.6057

**Published:** 2017-03-13

**Authors:** Chad Eric Darling, Silviu Dovancescu, Jane S Saczynski, Jarno Riistama, Fatima Sert Kuniyoshi, Joseph Rock, Theo E Meyer, David D McManus

**Affiliations:** ^1^ UMass Memorial Health Care Department of Emergency Medicine University of Massachusetts Medical School Worcester, MA United States; ^2^ Philips Research Eindhoven Netherlands; ^3^ Department of Pharmacy and Health Systems Sciences Northeastern University Boston, MA United States; ^4^ Philips Healthcare Andover, MA United States; ^5^ UMass Memorial Health Care Department of Medicine University of Massachusetts Medical School Worcester, MA United States

**Keywords:** telemedicine, outpatient monitoring, heart failure, electric impedance

## Abstract

**Background:**

Recurrent heart failure (HF) events are common in patients discharged after acute decompensated heart failure (ADHF). New patient-centered technologies are needed to aid in detecting HF decompensation. Transthoracic bioimpedance noninvasively measures pulmonary fluid retention.

**Objective:**

The objectives of our study were to (1) determine whether transthoracic bioimpedance can be measured daily with a novel, noninvasive, wearable fluid accumulation vest (FAV) and transmitted using a mobile phone and (2) establish whether an automated algorithm analyzing daily thoracic bioimpedance values would predict recurrent HF events.

**Methods:**

We prospectively enrolled patients admitted for ADHF. Participants were trained to use a FAV–mobile phone dyad and asked to transmit bioimpedance measurements for 45 consecutive days. We examined the performance of an algorithm analyzing changes in transthoracic bioimpedance as a predictor of HF events (HF readmission, diuretic uptitration) over a 75-day follow-up.

**Results:**

We observed 64 HF events (18 HF readmissions and 46 diuretic uptitrations) in the 106 participants (67 years; 63.2%, 67/106, male; 48.1%, 51/106, with prior HF) who completed follow-up. History of HF was the only clinical or laboratory factor related to recurrent HF events (*P*=.04). Among study participants with sufficient FAV data (n=57), an algorithm analyzing thoracic bioimpedance showed 87% sensitivity (95% CI 82-92), 70% specificity (95% CI 68-72), and 72% accuracy (95% CI 70-74) for identifying recurrent HF events.

**Conclusions:**

Patients discharged after ADHF can measure and transmit daily transthoracic bioimpedance using a FAV–mobile phone dyad. Algorithms analyzing thoracic bioimpedance may help identify patients at risk for recurrent HF events after hospital discharge.

## Introduction

It is estimated that over 25 million people worldwide suffer from heart failure (HF) [1,2]. Many patients with prior HF experience episodes of acute decompensated heart failure (ADHF) [3]. Both in Europe and in the United States, ADHF is responsible for nearly 1 million hospitalizations annually, and hospitalization rates from ADHF are increasing with the aging population [4,5,6]. In fact, ADHF is now the leading cause of hospital admissions among patients aged above 65 years in the United States [7].

Contemporary HF management programs rely on active surveillance for signs and symptoms of ADHF as well as medication-related and disease-specific education to optimize treatment. To date, these programs have shown only modest success. This is, in part, because commonly used clinical measures that include heart rate, blood pressure, and body weight poorly identify individuals at risk for subsequent HF events such as hospital admission [8,9]. There is, therefore, a need to develop new monitoring technologies to augment existing HF management programs. To be useful, these technologies must detect ADHF at an early stage, be acceptable to patients, facilitate communication between clinicians and patients, and relate to near-term HF decompensation and clinically relevant events.

Measures of thoracic bioimpedance, or opposition to an electric current through thoracic tissues, can be used to identify subclinical fluid retention [10]. Prior studies have demonstrated that intrathoracic bioimpedance measured in HF patients with implantable cardioverter-defibrillators (ICDs) predicts clinical events, including ADHF and HF-related hospitalizations [10,11]. As few patients with HF are eligible for an ICD, a noninvasive, user-friendly method for measuring thoracic bioimpedance would be scalable to a much more generalizable, broader population of patients. Small proof-of-concept studies have shown that a wearable, investigational device can measure thoracic bioimpedance, correlate with intrathoracic measures, and predict intrathoracic volume retention [12,13,14]. We designed the prospective SENTINEL-HF study to test the following two hypotheses in a cohort of patients recently discharged after hospitalization for ADHF: (1) Transthoracic bioimpedance can be measured daily with a novel, noninvasive, wearable fluid accumulation vest (FAV) and transmitted using a mobile phone and (2) an automated algorithm analyzing daily thoracic bioimpedance values would predict recurrent HF events.

## Methods

### Study Design and Setting

The rationale and design of the SENTINEL-HF study has already been published [15]. In brief, SENTINEL-HF is a prospective, nonrandomized, observational investigation including survivors of an admission for ADHF with New York Heart Association (NYHA) functional class II-IV HF. The final study sample consisted of adult patients hospitalized for ADHF at one of the 2 UMass-Memorial Medical Center (UMMMC) campuses between June 2013 and April 2015. The UMMMC serves a racially and sociodemographically diverse and elderly HF population in Central MA [16,17]. The 30-day all-cause readmission and death rates after a HF-related admission at UMMMC (23% and 12%, respectively) are similar to US national rates [18]. The study was approved by the Committee for the Protection of Human Subjects in Research at the University of Massachusetts Medical School (IRB H00001760) and was registered as a clinical trial (Clinicaltrials.gov NCT01877369).

### FAV and Mobile Phone App Description

**Figure 1:**
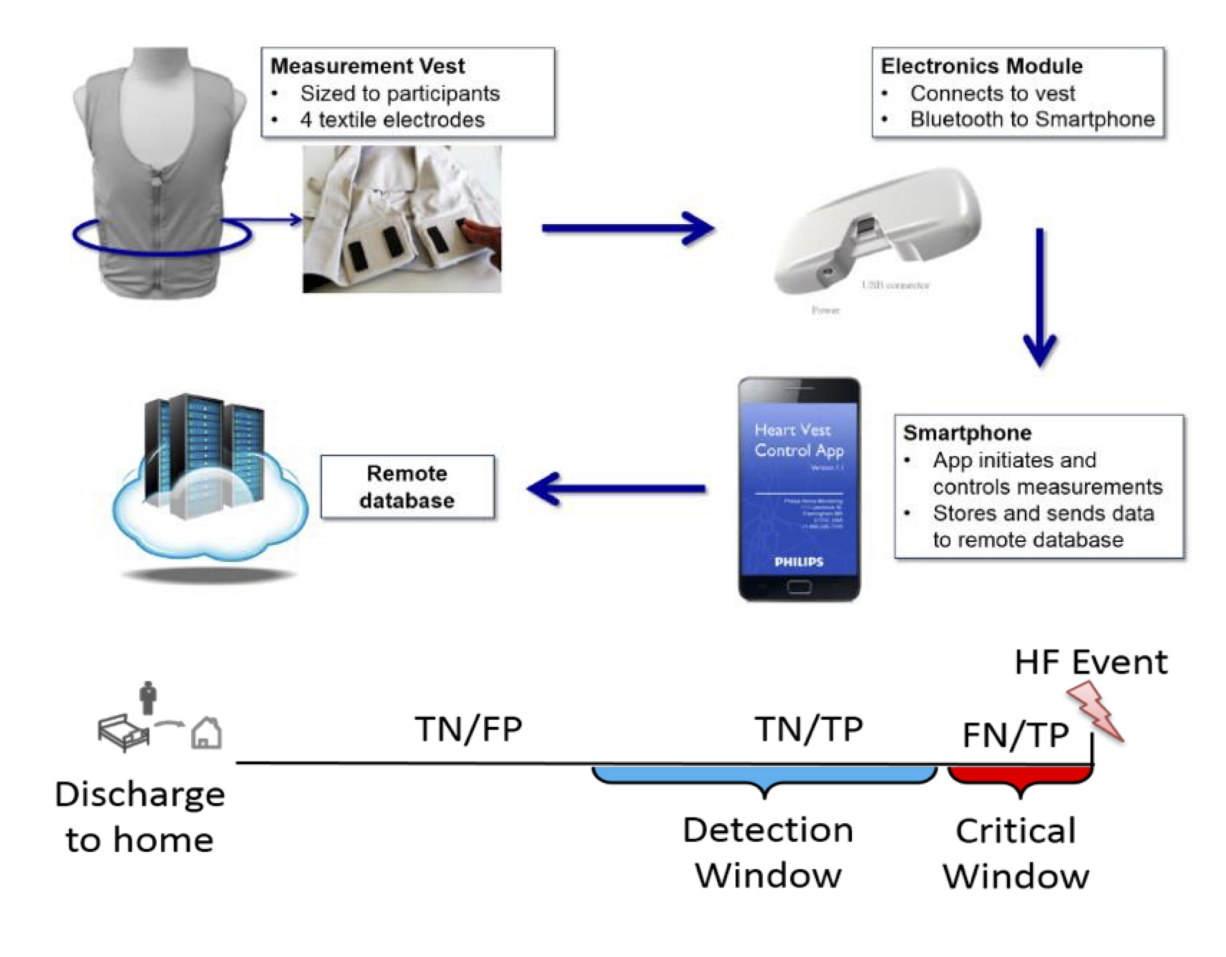
Components of the FAV and ADHF detection strategy. Top: components of the FAV measurement system and the process for data acquisition, transfer, and storage. Bottom: ADHF detection strategy. Depending on the presence of an alert, days are classified as TN or FP outside the detection window, TN or TP within the detection window, and FN or TP within the critical window. FAV: fluid accumulation vest; ADHF: acute decompensated heart failure; TN: true negative; TP: true positive; FN: false negative.

**Figure 2:**
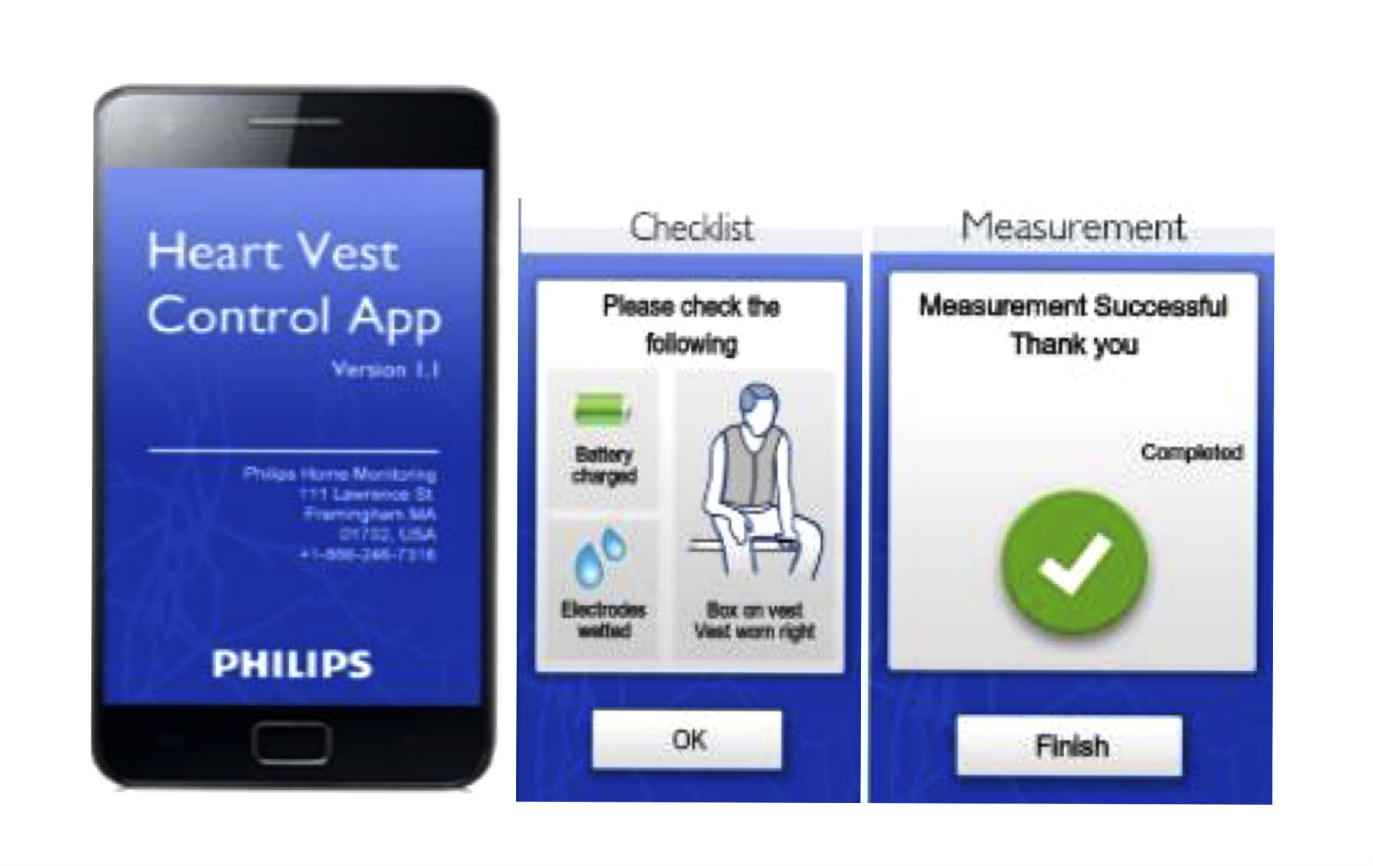
Representative images from the SENTINEL- HF study app running on a study mobile phone.

The FAV is a prototype, noninvasive wearable garment manufactured by Philips Inc, and designed to measure transthoracic bioimpedance from 4 reusable, embedded internal textile electrodes (Figure 1). The FAV is sized for each participant so that the electrodes rest snugly along the lower lateral aspect of the thorax. Unpublished work carried out by Philips Research before SENTINEL-HF has demonstrated acceptable precision of repeated bioimpedance measures using the FAV. An electronics module connected to the posterior aspect of the FAV enables wireless communication with a paired mobile phone app and determines transthoracic bioimpedance at multiple frequencies, enabling a Cole model-based assessment of intrathoracic fluid status [19]. The thoracic bioimpedance measurements are facilitated with a specially designed HF app running on an Android-based mobile phone (Figure 2). The phone-based app guides users through the measurement steps, controls measurement parameters, and transmits stored bioimpedance values to a secure cloud server using encoded GSM (Figure 2). The daily measurement routine requires an average of 8-10 min to complete.

### Study Procedures

SENTINEL-HF’s data collection was focused on 3 main activities (home monitoring using the FAV, participant interviews, and clinical event tracking) that were initiated during the index ADHF hospitalization and ended 75 days after hospital discharge. Trained study staff abstracted all relevant clinical information related to each subject’s index hospitalization, including medical history, physical exam data, radiographic findings, laboratory values, medications, as well as demographics from the medical record.

### Participant Recruitment

Patients with possible ADHF were identified using the hospitals’ electronic record by reviewing daily hospital admissions. Study staff determined SENTINEL-HF eligibility based on a review of admission diagnoses, chief complaint, and medical history. Eligibility was confirmed using laboratory reports, electrocardiographic data, and physical examination findings. Adult, English-speaking patients (age ≥21 years) were considered eligible for enrollment if they were admitted with a primary diagnosis of ADHF *or* if they had 1 sign (eg, vascular congestion on chest radiograph, any B-type natriuretic peptide level >100 ng/l) *and* 1 symptom (eg, dyspnea, orthopnea) consistent with ADHF. In addition, to ensure that only symptomatic HF patients were included, we restricted our inclusion to only those patients coded as having NYHA class II or greater by their examining physician. Participants and their caregivers also had to express a willingness to adhere to the study protocol for 45 days after hospital discharge. Prisoners, pregnant patients, patients with a permanent pacemaker or ICD, patients with a known nickel or electrode allergy, patients planning to move from their residence within 2 months of enrollment, as well as patients with any of the following diagnoses: end-stage renal disease requiring hemodialysis, home oxygen-dependent chronic obstructive pulmonary disease, severe primary pulmonary hypertension, severe psychiatric or neurological disorders, advanced cancer, or a body habitus preventing proper FAV electrode positioning, were considered ineligible for SENTINEL-HF. Patients were approached during their hospitalization and signed an informed consent.

Shortly before discharge, each enrolled participant was provided his or her own personalized FAV monitoring kit. Study staff demonstrated to participants (and their caregivers when possible) how to wear the FAV and use the mobile phone to obtain a measurement. Each mobile phone was programmed to automatically load a user-friendly app (Figure 2) that initiated an electronic handshake with the FAV, recorded a bioimpedance measurement, and transmitted stored bioimpedance measurements to a secure cloud-based server. After instruction, participants were asked to demonstrate proper use of the mobile phone and electronics module. They were provided a manual of operations, a short form of instructions, a troubleshooting guide, and a magnet with the study staff contact information.

### Home Monitoring Protocol

**Figure 3:**
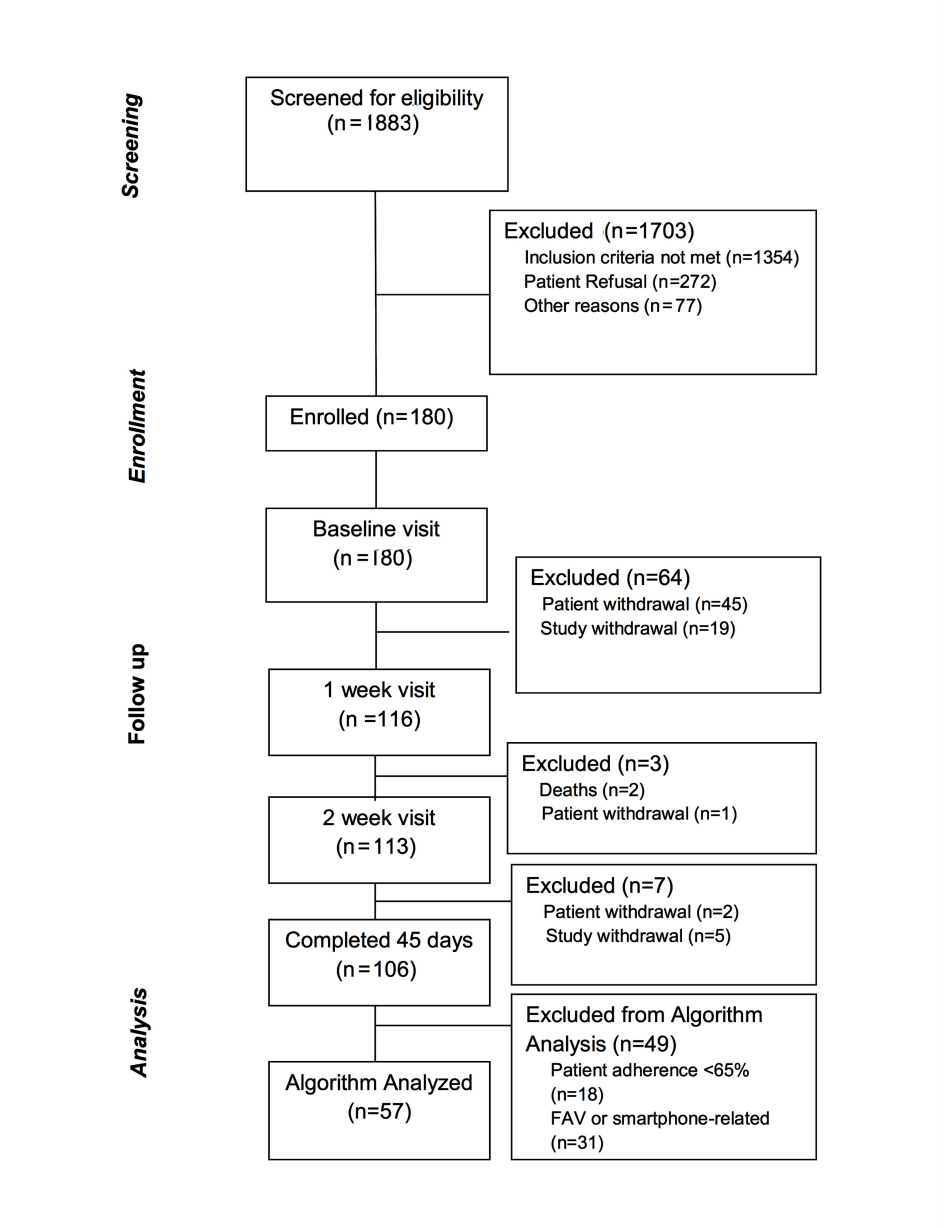
Participant flow diagram for the SENTINEL-HF study.

During a 45-day home monitoring period, participants were required to perform daily 5-min FAV measurements sitting upright in a chair at a consistent time each morning. On postdischarge day 1, the study staff called each participant to answer questions related to the first FAV assessment. Participant adherence was monitored on a daily basis and tracked based on transmissions to the remote database. Study staff called participants with 2 or more consecutive missed or nonevaluable measurements. If 2 subsequent missed days occurred after the call, study staff visited the participant at home to troubleshoot and provide a FAV self-assessment walk-through. As shown in Figure 3 participants were removed from the study if 2 consecutive missed days occurred after the home visit.

### Participant Interviews

Participants were interviewed in person or over the telephone at enrollment (as an inpatient) and at days 7, 14, and 45 after discharge. During these follow-up interactions, key patient reported outcomes were assessed. Validated instruments included the Kansas City Cardiomyopathy Questionnaire (KCCQ) [20] and the Telephone Interview for Cognitive Status (TICS) [21]. Participants were also asked about medication changes, health care visits, or hospital readmissions. Reports were confirmed by reviewing the medical record.

### Study Outcomes and Endpoint Adjudication

This study assessed the ability of an automated algorithm that evaluated daily transthoracic bioimpedance measures to predict a composite endpoint of unplanned, HF-related rehospitalization or diuretic uptitration over a total of 75 days following hospital discharge. Endpoints were adjudicated on a rolling basis by 3 physicians (TEM, DDM, and CD) blinded to FAV measures and clinical outcomes. FAV measures were not used for clinical management, as this was an observational study.

Passive surveillance for study endpoints after hospital discharge was carried out by review of clinical data, including hospital visits, clinic notes, cardiac studies, laboratory reports, operative reports, consultations, and hospital discharge summaries. As most (>80%) patients hospitalized for ADHF at UMMMC follow-up with a UMMMC cardiologist and are readmitted at UMMMC, the vast majority of participant data was available in one centralized electronic medical record. Information related to patient-reported clinical events occurring outside our hospital system was obtained from the respective health care providers or from the outside medical record.

### HF Decompensation Detection Algorithm

**Figure 4:**
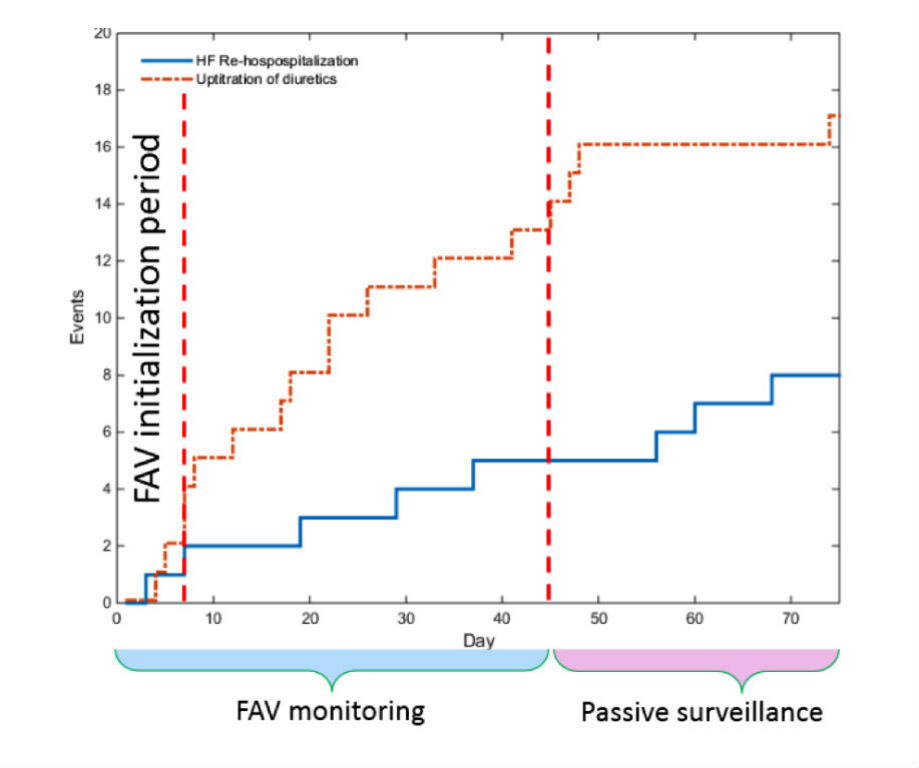
Cumulative clinical HF events over the 75-day follow-up period. Red line = Diuretic up-titrations, Blue Line = HF Rehospitalizations. FAV learning period = first 6 days, FAV monitoring period = next 39 days. Total surveillance period = 75 days. HF: heart failure; FAV = fluid accumulation vest.

The ADHF detection algorithm is tailored to each patient, and it monitors the daily evolution of transthoracic bioimpedance index (Figure 4). Specifically, the algorithm classifies every impedance measurement as “normal” or “abnormal” using an adaptive range around the expected normal bioimpedance for the patient. The ”normal” range for a given patient is established over the first 4 days after discharge using the patient’s initial measurements and knowledge from prior studies about the normal variability of the bioimpedance index in ambulatory, asymptomatic HF patients^.^ [17,22,23]. The algorithm raises a HF alert if it notes a sustained reduction in bioimpedance index, as determined by 3 consecutive days of bioimpedance values below the patient-specific normal range. Therefore, the earliest possible HF alert occurred on day 7 after discharge.

### Data Analysis

Our study sample was divided into 2 groups for analysis: (1) participants with a clinically relevant recurrent HF event (HF-related readmission or diuretic uptitration) during the 75-day follow-up period; and (2) participants without clinical HF events. To compare participants with and without events, we calculated frequencies and percentages for nominal categorical variables, means and standard deviations for continuous variables, and mean and interquartile ranges for ordinal variables, for all patients and for each of the 2 groups separately. We compared differences between the 2 groups by using the chi-square test for nominal categorical variables with cell size ≥5 and Fisher exact test for variables with small cell size (<5). Differences in means between the 2 groups were compared using *t* test and nonparametric Wilcoxon test. The Wilcoxon test was also used to compare differences in medians between ordinal variables. A *P* value of <.05 was considered significant.

To analyze the performance of the automated HF detection algorithm, we restricted our analysis to subjects with sufficient usable bioimpedance data (defined as ≥65% analyzable, daily bioimpedance measurements). We first compared the characteristics of participants with and without usable bioimpedance data using the methods described above. We then examined the predictive ability of the FAV by calculating sensitivity, specificity, and accuracy of the HF detection algorithm with the following definitions and assumptions: First, fluid accumulation due to worsening HF may begin several days to weeks before a clinically apparent HF event. We defined the time window between the period when a decompensation event begins and the occurrence of a clinically apparent HF event as the detection window. We then defined the 3 days before any HF event as the critical window. We set the critical window at 3 days based on two assumptions: (1) fluid accumulation leading to a HF decompensation would be highly likely to be present during this period, and (2) 3 days would provide the minimum actionable period for a clinician to receive an alert and act on it (Figure 1).

Each monitoring day was then classified as a true positive (TP), true negative (TN), false positive (FP), or false negative (FN). We considered a TP any day when an alert was present and preceded a HF event within a 30-day detection window. Any day with an alert raised outside the 30-day detection window was considered an FP. Each day without an alert outside the critical window was considered a TN. Finally, all days without an alert but within the critical window were considered FNs (Figure 1 **)**. A sensitivity analysis was carried out by varying the lengths of the critical window from 3 to 14 days and the detection window from 14 to 30 days. All analyses were conducted using SAS 9.3 (SAS Institute) and MATLAB 2014b (MathWorks).

## Results

### Study Participants

Of the 180 participants who consented to participate in SENTINEL-HF, 106 completed the 75-day follow-up period (Figure 3). Patient request to leave the study (n=45), for a variety of reasons, was the most common cause for early dropout. As shown in Table 1, the average age of study participants was 67 years, 63% were male, and 91% were white. The burden of comorbid cardiovascular risk factors and diseases was high, disorders of cognition were present in nearly a third of patients, and HF symptom severity was modest. Of the 106 participants who completed follow-up, participants with a history of HF (*P*<.05) were more likely to have a recurrent HF event than were patients without known HF at the time of their ADHF admission. Notably, length of index hospital stay, peak B-natriuretic peptide level, left ventricular systolic function, and age were not related to hospital readmission within 75 days.

**Table 1:** Characteristics of 106 SENTINEL-HF participants with and without recurrent heart failure (HF) events during the 75-day follow-up.

Demographic characteristic		All (n=106)	Without recurrent HF event (n=61)	With recurrent HF event (n=45)	*P* value^a^
Age (years), mean (SD)^b^		67.2 (11.2)	67.7 (10.7)	66.4 (12.0)	.52
Male, n (%)		67 (63.2)	37 (61)	30 (67)	.53
Race (white), n (%)		96 (90.6)	58 (95)	38 (84)	.06
Body mass index (kg/m^2^), mean (SD)		32.9 (7.6)	32.5 (8.0)	33.4 (7.0)	.48
Left ventricular Ejection fraction, mean (SD)		44.0 (18.3)	43.6 (18.0)	44.1 (19.1)	.85
NYHA class^c^, median (IQR)^b^		3 (2-4)	3 (2-4)	3 (2-4)	.19
**Index admission, mean (SD)**					
	BNP^d^, ng/dL	864.0 (782.7)	980.41 (938.9)	708.0 (472.8)	.66
	Serum creatinine (mg/dL)	1.2 (0.6)	1.2 (0.5)	1.4 (0.7)	.07
	Serum sodium (mg/dL)	136.0 (3.4)	136.4 (3.4)	136.4 (3. 6)	.85
	Systolic blood pressure (mmHg)	136.0 (27.0)	135.7 (28.2)	135.4 (25.54)	.77
	Diastolic blood pressure (mmHg)	79.0 (19.9)	79.9 (17.9)	77.0 (22.3)	.29
	Heart rate (beats/min)	91.0 (24.5)	93.3 (24.1)	89.0 (25.1)	.35
	Respiratory rate (breaths/min)	20.0 (4.2)	20.1 (4.6)	20.3 (3.7)	.57
	KCCQ^e^ total symptom score	45.0 (26.1)	45.1 (25.8)	43.9 (26.8)	.85
	TICS^f^ total score	32.0 (4.2)	32.6 (3.5)	31 (4.9)	.08
**Hospital characteristics, mean (SD)**					
	Hospital length of stay (days)	4.0 (3.7)	5.5 (3.6)	5 (3.1)	.53
**Medical history, n (%)**					
	Myocardial infarction	22 (20.7)	10 (16)	12 (27)	.20
	Coronary artery bypass	17 (16.0)	7 (11)	10 (22)	.14
	Hypertension	85 (80.2)	50 (82)	35 (78)	.59
	Stroke	4 (3.8)	3 (5)	1 (2)	.64
	Heart failure	51 (48.1)	24 (39)	27 (60)	.04
	Diabetes mellitus	48 (5.2)	24 (39)	24 (53)	.15
	Hypercholesterolemia	53 (50.0)	32 (52)	21 (47)	.56
	Chronic lung disease	23 (21.7)	14 (23)	9 (20)	.72
	Renal failure	15 (14.1)	7 (11)	8 (18)	.36
	Atrial fibrillation	36 (33.9)	18 (30)	18 (40)	.26
**Home medications, n (%)**					
	Beta blockers	87 (82.1)	49 (80)	38 (84)	.59
	ACE^g^-inhibitors	63 (59.4)	40 (66)	23 (51)	.13
	Diuretics	93 (87.7)	55 (90)	38 (84)	.37
	Digoxin	14 (13.2)	9 (15)	5 (11)	.58

^a^*P* value compares the subgroups with HF events (HF rehospitalization or diuretic uptitration).

^b^Data are expressed as mean (SD) for continuous variables, and median (IQR) for ordinal variables. Counts are reported as percentage of the respective subgroup.

^c^NYHA: New York Heart Association.

^d^BNP: B-type natriuretic peptide.

^e^KCCQ: Kansas City Cardiomyopathy Questionnaire.

^f^TICS: telephone interview for cognitive status.

^g^ACE: Angiotensin-converting enzyme.

**Figure 5:**
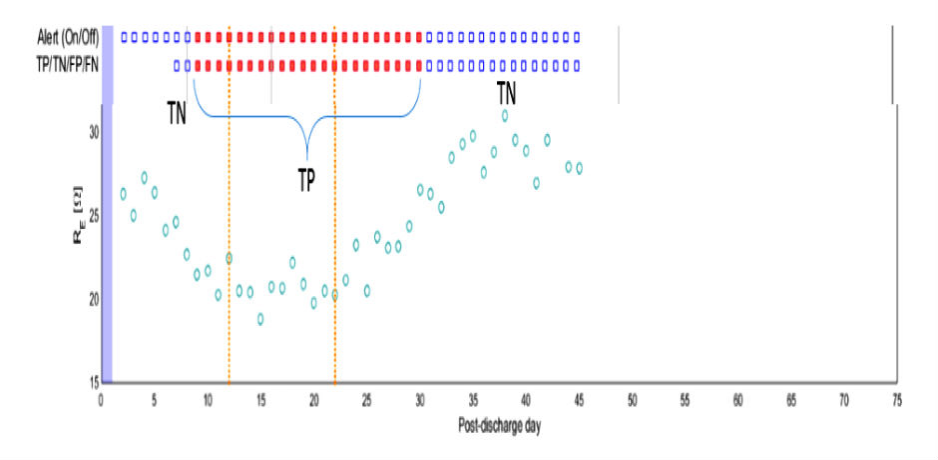
Daily bioimpedance values, HF alerts, and recurrent HF events from a representative SENTINEL-HF participant. Serial bioimpedance measurements (circles) in a SENTINEL-HF participant with 2 clinical HF events (gold lines). This participant experienced a decrease in bioimpedance starting 1 week after hospital discharge. The participant’s diuretic dose was increased twice (vertical lines) due to increased edema and bioimpedance measures returned toward the baseline. The red dots indicate daily FAV alerts. HF: heart failure; FAV = fluid accumulation vest.

### Data Acquisition and Protocol Adherence

When supported by the study staff, approximately half (n=87) of the 180 consenting participants transmitted usable bioimpedance measures on over 65% of days during the 45-day follow-up. The major reason for study withdrawal was nonadherence to scheduled FAV uploads for >2 consecutive days. In addition to patient-initiated study withdrawal, technical factors, including motion and noise artifact, or mobile phone app malfunction, contributed to the data loss (n=30). This left 57 participants (53.8%), with sufficient bioimpedance data for the automated HF detection algorithm to analyze (Figure 3). The characteristics of the SENTINEL-HF participants with adequate interpretable data did not vary significantly from those who did not have sufficient data, with the exception of a slightly longer index hospital stay observed among patients with lower adherence to the FAV (Table 2).

**Table 2:** Characteristics of SENTINEL-HF participants who completed follow-up, further stratified by inclusion or exclusion in algorithm analyses.

Characteristics		All (n=106)	Included (n=57)	Excluded (n=49)	*P* value^a^
Age (years), mean (SD)^b^		67.2 (11.2)	66.8 (9.9)	67.65 (12.7)	.77
Male, n (%)		67 (63.2)	34 (60)	33 (67)	.41
Race (white), n (%)		96 (90.6)	51 (89)	45 (92)	.68
Body mass index (kg/m^2^), mean (SD)		32.9 (7.6)	32.0 (6.7)	34.0 (8.4)	.22
LV ejection fraction, mean (SD)		44.0 (18.3)	44.1 (19.0)	43.5 (17.8)	.94
NYHA^c^ class, median (IQR)^b^		3 (2-4)	3 (2-4)	3 (2-3)	.71
**Index admission, mean (SD)**					
	BNP^d^ (ng/dL)	864.0 (782.7)	997 (967)	712.5 (465.5)	.48
	Serum creatinine (mg/dL)	1.2 (0.6)	1.2 (0.5)	1.3 (0.7)	.24
	Serum sodium (mg/dL)	136.0 (3.4)	137 (2.8)	136.0 (4.1)	.68
	Systolic blood pressure (mmHg)	136.0 (27.0)	135.3 (29.3)	136.0 (24.3)	.60
	Diastolic blood pressure (mmHg)	79.0 (19.9)	78.8 (20.2)	78.5 (19.7)	.73
	Heart rate (beats/min)	91.0 (24.5)	90.5 (22.9)	92.5 (26.6)	.81
	Respiratory rate (breaths/min)	20.0 (4.2)	20.1 (5.0)	20.3 (3.1)	.22
	KCCQ^e^ total symptom score	45.0 (26.1)	49.0 (26.2)	39.3 (25.2)	.07
	TICS^f^ total score	32.0 (4.2)	32.2 (3.6)	31.6 (4.8)	.71
**Outcome, n (%)**					
	HF readmission over 75 days	14 (13.2)	6 (11)	8 (16)	.38
	Diuretic uptitration over 75 days	36 (33.9)	15 (26)	21 (43)	.07
**Medical history, n (%)**					
	Myocardial infarction	22 (20.7)	12 (21)	10 (20)	.94
	Coronary artery bypass	17 (16.0)	7 (12)	10 (20)	.26
	Hypertension	85 (80.2)	46 (81)	39 (80)	.89
	Stroke	4 (3.8)	1 (2)	3 (6)	.24
	Heart failure	51 (48.1)	27 (47)	24 (49)	.87
	Diabetes mellitus	48 (5.2)	25 (44)	23 (47)	.75
	Hypercholesterolemia	53 (50.0)	33 (58)	20 (41)	.08
	Chronic lung disease	23 (21.7)	11 (19)	12 (25)	.52
	Renal failure	15 (14.1)	7 (12)	8 (16)	.55
	Atrial fibrillation	36 (33.9)	15 (26)	21 (43)	.07
**Home medications, n (%)**					
	Beta blockers	87 (82.1)	47 (82)	40 (82)	.91
	ACE^g^-inhibitors	63 (59.4)	35(61)	28 (57)	.66
	Diuretics	93 (87.7)	49 (86)	44 (90)	.55
	Digoxin	14 (13.2)	7(12)	7 (14)	.76

^a^*P* value compares the subgroups with HF events (HF rehospitalization or diuretic uptitration).

^b^Data are expressed as mean (standard deviation) for continuous variables, and median (interquartile range) for ordinal variables. Counts are reported as percentage of the respective subgroup.

^c^NYHA: New York Heart Association.

^d^BNP: B-type natriuretic peptide.

^e^KCCQ: Kansas City Cardiomyopathy Questionnaire.

^f^TICS: telephone interview for cognitive status.

^g^ACE: Angiotensin-converting enzyme.

### Study Endpoints

Among the 57 participants with ADHF who were included in our analysis of the HF detection algorithm, we observed 25 HF events in 20 patients over a 75-day follow-up. HF events included 8 HF-related hospital readmissions and 17 diuretic uptitrations. HF events were evenly distributed throughout the 75-day follow-up period (Figure 4).

The mean time between a bioimpedance-based HF alert and a clinical HF event was 21 days (median 11 days). Figure 5 illustrates a representative participant’s daily bioimpedance and the relationship among impedance changes, HF alerts, and HF events. The automated HF prediction algorithm exhibited reasonable sensitivity but modest specificity for prediction HF events within the 30 days of an alert with a sensitivity of 87% (95% CI 82-92), specificity of 70% (95% CI 68-72), and accuracy of 72% (95% CI 70-74; Table 3). A sensitivity analysis (Table 3) examining the effect of varying the detection window and critical window showed that shortening the detection window from 30 days or expanding the critical window beyond 3 days reduced the predictive performance of the automated HF detection algorithm.

**Table 3:** Sensitivity analysis for fluid accumulation vest (FAV) performance.

			
**Detection window, days**		14 days				21 days				30 days		
**Critical window, days**		3	7	14		3	7	14		3	7	14
**Participants**												
	Total number	57	57	57		57	57	57		57	57	57
	Subjects with HF events	16	16	16		16	16	16		18	18	18
**HF detection performance**												
	Sensitivity (%)	82	66	50		84	70	55		87	74	61
	Specificity (%)	70	69	68		70	70	69		70	70	69
	Accuracy (%)	70	69	66		71	70	67		72	70	68

## Discussion

### Principal Findings

The results of this prospective, observational study demonstrate that older patients recently discharged after a HF event can measure and transmit transthoracic bioimpedance daily using a novel vest–mobile phone system. Our findings also show that an automated algorithm analyzing transthoracic bioimpedance was able to detect HF decompensation with reasonable sensitivity before clinical HF events. Automated bioimpedance measurement and analysis is possible using mobile technologies and may provide an innovative mechanism to monitor HF patients and improve their care.

As patients who have had a recent HF-related hospitalization are known to be at high risk for recurrent HF decompensation and readmission after discharge [22], we recruited participants admitted with ADHF. Readmissions after a HF hospitalization are often considered preventable and reflective of poor in-hospital management or discharge practices [3]. In recent years, the Patient Protection and Affordable Care Act established payment reforms that penalize US hospitals with high readmission rates following a HF-related hospitalization [24]. HF admissions are associated with great personal cost to the patient and economic costs to our health care system [25,26], and readmission penalties have heightened interest in developing innovative HF management programs. As traditional physical exam findings have been found to be unreliable predictors of ADHF in patients with chronic HF [27], novel technologies are needed to help identify HF decompensation. Moreover, monitoring patients with HF at home is vital as it has been demonstrated that ambulatory HF patients need guidance in order to seek care in a timely fashion [27-29].

The FAV is designed to measure thoracic bioimpedance, which varies with the patient-specific fluid volume. Several studies have associated thoracic fluid accumulation, with HF decompensation. These measures compare favorably with clinical HF metrics such as weight gain [30-33]. In the SENSE-HF trial, a study of 501 ambulatory patients with chronic HF [34] and an ICD, intrathoracic bioimpedance exhibited modest sensitivity (up to 42%) for predicting HF-related readmissions. Although some efforts to use continuous bioimpedance values coupled with patient-agnostic cut-offs for determining abnormal values have exhibited suboptimal accuracy [34], recent studies have demonstrated that more sophisticated approaches improve the performance of automated algorithms analyzing bioimpedance [30-33]. On this basis, many clinical HF programs use intrathoracic bioimpedance to guide therapeutic decision making in ambulatory patients with chronic HF and implantable defibrillators [35,36]. Although bioimpedance assessment using implantable devices is helpful for many HF patients with severely reduced systolic dysfunction, the majority of HF patients do not have them.

Therefore, we tested a new approach using a wearable device paired to a mobile phone to monitor thoracic bioimpedance as such systems have shown promise in preliminary studies [12] and would be potentially usable by a more diverse and larger number of ambulatory patients with HF. Moreover, we also sought to improve on existing analytic approaches by training our algorithm to familiarize itself with a patient-specific normal bioimpedance range.

Overall, when supported through in home visits and phone instructions, about half of the study participants transmitted usable bioimpedance measures on over 65% of study days during the postdischarge period. Participants were able to achieve a reasonable adherence rate to this intensive program despite being older, symptomatic, and having a modest burden of psychosocial and cognitive impairments. We had to withdraw 49 participants (Table 2) from the study primarily due to nonadherence to scheduled uploads. Only 30 participants had data that were deemed “nonusable,” and the proportion of individuals with nonusable data decreased over time, as several factors such as motion and noise artifact or mobile phone FAV pairing errors (ie, not syncing or defaulting to airplane mode) were identified and corrected by study engineers and staff who reviewed data on a daily basis. We observed that daily review of uploads was helpful to improve data quality and target participants struggling with device use. Other studies have had similar adherence issues to telemonitoring with ranges of adherence between 4% and 55% withdrawing after enrollment [37]. Although we did not observe significant differences between study participants excluded for nonadherence and those who were able to adhere to FAV use, further research is needed to investigate factors associated with adherence to enable ideal targeting of this monitoring strategy.

Clinical HF recurrence was common in our cohort but consistent with rates reported in prior studies of patients hospitalized for ADHF [17]. Despite the aforementioned loss of some device data due to corruption or loss of transmission, our automated algorithm analyzing transmitted thoracic bioimpedance values demonstrated reasonable performance as a screening test for participants with usable data, with a sensitivity of 87%, specificity of 70%, and an overall accuracy of 72%. In a prior study including 33 patients with NYHA class III-IV HF who underwent implantation of a HF monitoring device and were followed for a mean 21 (SD 8) months, intrathoracic bioimpedance correlated strongly and inversely with pulmonary capillary wedge pressure and net fluid loss. In this same study, automated detection of decreased intrathoracic impedance showed somewhat lower sensitivity (77%) for predicting rehospitalizaton for HF compared with that observed in our study [38]. In another study including 43 older, ambulatory HF patients with an ICD capable of intrathoracic bioimpedance measurement, and followed over an average of 220 days for worsening HF (as defined by hospitalization or signs or symptoms of HF), changes in bioimpedance had a positive predictive value of 78.6% [39]. Finally, the SENSE-HF trial showed in 501 older participants with HF and an ICD capable of intrathoracic bioimpedance measurement that sensitivity was low during the 6 months after device implant, but improved 6 months after implantation [34]. In sum, our results suggest that transthoracic bioimpedance-based HF decompensation detection shows similar performance to automated intrathoracic biompedance-based approaches [10,34,39].

As exhibited by our sensitivity analyses, the HF detection algorithm performance varied when the duration of the detection window or critical window were altered. Our sensitivity analysis also highlights an important aspect of any bioimpedance telemonitoring system for HF patients. Our data suggests that individual patterns of bioimpedance variability exist, and therefore in the days leading up to a HF decompensation alerts may be constant or unpredictable and intermittent. Therefore, setting “rules” for how a bioimpedance algorithm would be expected to perform in a heterogenous group of patients is difficult, and further study and refinement of the detection algorithm may lead to greater accuracy. With additional optimization, FAV measurements may provide key data as part of an overall home care system that monitors a range of objective (eg, changes in weight and vital signs) and subjective factors (eg, HF symptoms).

A variety of systems have been put in place to support patients with HF in the outpatient setting [8,40]. These programs typically serve the dual function of supporting the patient with HF at home so they can better manage their HF, and secondarily they seek to monitor the clinical status of each patient [41,42]. However, monitoring for signs and symptoms of HF, including analyses of symptom scores, blood pressure, heart rate, and weight, perform poorly as predictors of clinically relevant HF events [3,42]. Following daily thoracic bioimpedance might augment such systems by providing a more specific measure of intrathoracic fluid.

As many wearables, including the FAV, collect electrocardiographic and respiratory data, in addition to bioimpedance [43], we anticipate that bioimpedance analysis will ultimately be integrated into a multiparameter HF monitoring approach. We also expect that our mobile phone app might be enhanced to enable daily symptom assessment or documentation of medication usage. We envision that implementation of the FAV-mobile phone monitoring system in a clinical system should occur in the context of a comprehensive HF management program, be guided by HF specialists, and be targeted at patients with recurrent HF admissions who are not ICD candidates. Comprehensive HF programs have the advantage of longitudinal relationships with HF patients that extend beyond inpatient service lines and into the clinic, expertise monitoring HF patients for decompensation using bioimpedance, and knowledge needed to guide therapeutic changes to improve quality of life and reduce HF readmissions, and thus are well-suited to perform transthoracic bioimpedance monitoring using existing infrastructure.

### Study Strengths and Limitations

There are several strengths of this study. First, it included the enrollment of a generalizable, older cohort of participants with ADHF and multiple psychosocial, cognitive, and cardiovascular comorbidities. Second, our study utilized rigorous and blinded adjudication of clinically relevant HF events. A third strength was our use of validated patient-reported assessments to phenotype study participants vis-a-vis their cognitive status, psychosocial characteristics, and symptom-burden. However, several limitations should be considered when interpreting the results of this study. First, the overall sample size was modest due to data loss and patient withdrawal. A prespecified interim analysis for sample size reassessment revealed that technical factors, including motion and noise artifact and mobile phone app malfunction, contributed to data loss. To help mitigate this, we implemented a feedback loop to assess measurement quality and recommend corrective actions, which enhanced the amount of interpretable data. Notably, participants included in the HF detection algorithm analysis did not differ significantly from participants without sufficient interpretable data, enhancing generalizability. Second, our specificity was affected by FPs. The FP rate is influenced by many factors including the time interval considered relevant for tracking bioimpedance before a HF event. We chose to have patients only do a single daily measurement, but taking additional measures may have decreased the FP rate. The other factor affecting FPs relates to the target sensitivity of the FAV system. If a lower sensitivity was targeted, the specificity would have risen at the expense of missed HF exacerbations that may be unacceptable. Third, our cohort was racially homogenous and from a single US region. The generalizability of our findings to other racial and ethnic groups with HF is therefore unclear. Finally, FAV support by the study staff might limit our generalizability to situations where inhome or telephone support may not be feasible. Nevertheless, our approach was not greatly different from some intensive clinical HF monitoring programs and may provide a blueprint for future clinical HF programs that incorporate novel technologies for HF assessment.

### Conclusions

We observed that an automated algorithm analyzing daily bioimpedance uploads from a FAV–mobile phone dyad predicted clinically relevant HF recurrences in a cohort of older survivors of an ADHF-related hospitalization, who were followed for 75 days postdischarge. We are heartened by these findings but recognize that enhancements are needed to improve the percentage of usable bioimpedance data, perhaps through real-time analysis and feedback to participants with noisy or uninterpretable data uploads. Future clinical trials should focus on enhancing the accuracy of ADHF prediction by assessing additional parameters of cardiovascular health and should evaluate whether bioimpedance-informed therapeutic decisions reduce HF events. We are optimistic that mobile HF monitoring solutions such as ours will ultimately improve the prognosis and quality of life of ambulatory patients with chronic HF.

## Abbreviations

ADHF: acute decompensated heart failure
FAV: fluid accumulation vest
FP: false positive
FN: false negative
HF: heart failure
ICD: implantable cardioverter-defibrillator
KCCQ: Kansas City Cardiomyopathy Questionnaire
TICS: Telephone Interview for Cognitive Status
TP: true positive
TN: true negative
